# Predicted Excess Cardiovascular Age and a Reverse Socioeconomic Gradient in a Middle-Income Latin American Country: A Population-Based Analysis of 163,889 Peruvians

**DOI:** 10.3390/jcdd13070318

**Published:** 2026-07-09

**Authors:** Víctor Juan Vera-Ponce, Jhosmer Ballena-Caicedo, Jhofree Einstein Briceño-Chavez, Kevin Cusma-Regalado, Fiorella E. Zuzunaga-Montoya, Julio César Bautista Zuta, Rossmery Leonor Poemape Mestanza

**Affiliations:** Facultad de Medicina (FAMED), Universidad Nacional Toribio Rodríguez de Mendoza de Amazonas (UNTRM), Chachapoyas 01001, Amazonas, Peru; 7330178022@untrm.edu.pe (J.B.-C.); 7268018622@untrm.edu.pe (J.E.B.-C.); 7173968722@untrm.edu.pe (K.C.-R.); fiorella.zuzunaga@untrm.edu.pe (F.E.Z.-M.); julio.bautista@untrm.edu.pe (J.C.B.Z.); rossmery.poemape@untrm.edu.pe (R.L.P.M.)

**Keywords:** vascular health, health inequalities, Slope Index of Inequality, concentration index, cardiovascular diseases, Peru

## Abstract

Predicted cardiovascular age (heart age) translates the risk-factor profile into an equivalent age, which may facilitate interpretation of estimated cardiovascular risk. Excess cardiovascular age describes, in years, the integrated burden of modifiable risk factors and its distribution in the population. This study aimed to quantify socioeconomic and geographic inequalities in predicted excess cardiovascular age among Peruvian adults using standardized inequality measures, and to describe its temporal variation from 2014 to 2024. We analyzed ENDES Peru 2014–2024 data for adults aged 30–74 years. Cardiovascular age was estimated using the body mass index (BMI)–based non-laboratory Framingham equation, and excess was defined as the difference between cardiovascular age and chronological age. Weighted means and 95% confidence intervals were estimated accounting for the complex survey design. Socioeconomic inequalities were assessed using absolute and relative gaps between extreme wealth quintiles (Q5–Q1), the Slope Index of Inequality (SII), the Relative Index of Inequality (RII), and the concentration index/curve. Among 163,889 participants, mean excess cardiovascular age was 9.64 years (95% CI: 9.48–9.80), with similar estimates in women (9.73; 95% CI: 9.52–9.94) and men (9.54; 95% CI: 9.33–9.75). Temporal variation was observed, peaking in 2021 (10.91; 95% CI: 10.57–11.25). Excess increased with wealth (Q1: 7.14 vs. Q5: 11.25 years), with an SII of 5.04 years (95% CI: 4.71–5.37) and a concentration index of 0.087. The gradient was steeper in men (SII 6.14) than in women (SII 3.90). Geographically, Metropolitan Lima had higher excess than the Highlands (11.17 vs. 7.45 years), and urban areas exceeded rural areas (10.28 vs. 7.25 years). In Peru, adults aged 30–74 years had a mean predicted excess cardiovascular age of about 10 years, with a consistent pro-rich and urban/coastal concentration pattern, more pronounced among men. Because this metric is derived from a risk prediction equation, these findings should be interpreted as surveillance-oriented evidence of inequalities in estimated risk-factor burden, not as evidence of observed cardiovascular disease, subclinical cardiovascular damage, causal mechanisms, or tested intervention effects.

## 1. Introduction

Cardiovascular diseases (CVD) are the leading cause of death and one of the largest sources of lost healthy life years worldwide. Evidence from the Global Burden of Disease shows that the burden of CVD has increased since 1990, driven by population ageing and the persistence of modifiable risk factors such as elevated blood pressure and dietary risks [1].

The cardiovascular burden is unequally distributed within and between countries. In particular, in low- and middle-income countries, socioeconomic position shapes exposure to risk factors, access to prevention and treatment, and continuity of care, contributing to avoidable gaps in cardiovascular morbidity and mortality [2]. However, the social gradient is not static: its direction and intensity can vary depending on the stage of epidemiological transition and the level of income. The multinational PURE study showed that the relationship between socioeconomic status, risk factors, and cardiovascular events differs across countries at different development levels, suggesting that structural determinants and health-system responses modulate observed inequalities [3].

In Latin America and the Caribbean, the epidemiological and nutritional transition has been rapid and heterogeneous, with changes in the socioeconomic concentration of risk factors. Obesity, for example, has gradually shifted toward a higher burden in less advantaged strata as the obesity transition advances in the region [4]. In Peru, urbanization and demographic changes coexist with territorial heterogeneity and persistent inequalities, underscoring the need for population metrics that can identify higher-risk groups and guide equitable preventive interventions [5].

Current guidelines recommend estimating cardiovascular risk and emphasize effective risk communication as an integral component of prevention [6]. Nevertheless, absolute risk can be unintuitive, and alternative formats have been proposed. Predicted cardiovascular age (“heart age”) expresses risk in years and has recently been estimated in multiple countries using representative surveys, showing that a substantial proportion of adults have a cardiovascular age higher than their chronological age across diverse settings [7]. Still, evidence on its impact on behavioral or clinical change is heterogeneous: a recent meta-analysis suggests modest and variable effects depending on format and context [8].

Although cardiovascular age has been estimated in multinational surveys, evidence remains limited on how predicted excess cardiovascular age is distributed across socioeconomic strata and subnational territories in Peru using standardized inequality metrics and repeated nationally representative surveys. The contribution of this study is therefore surveillance-oriented: to quantify the distribution of an interpretable, risk-factor–based cardiovascular metric and its socioeconomic and geographic inequalities, rather than to validate the Framingham equation, estimate observed cardiovascular disease incidence, or infer causal mechanisms. We analyzed ENDES 2014–2024 data from adults aged 30–74 years to estimate predicted excess cardiovascular age and quantify inequalities by wealth, education, area of residence, natural region, and department.

## 2. Materials and Methods

### 2.1. Study Design

This study is a secondary analysis of microdata from the Demographic and Family Health Survey (ENDES) of Peru, covering multiple annual rounds from 2014 to 2024. We used an observational approach based on repeated cross-sectional surveys to estimate the population distribution of excess cardiovascular age and to quantify inequalities by socioeconomic conditions and geographic domains. The manuscript was prepared following the STROBE reporting guideline for observational studies [9].

### 2.2. Data Source

ENDES is a population-based survey conducted by the National Institute of Statistics and Informatics (INEI), designed to produce estimates representative at the national level and by geographic domains. The survey uses a complex sampling design: two-stage, probabilistic, stratified, and independent at the departmental level and by urban/rural area. For this study, we used microdata from the Health Questionnaire applied to one selected household member aged 15 years or older, which includes blood pressure and anthropometric measurements, as well as information relevant to the cardiometabolic profile [10].

### 2.3. Study Population and Eligibility Criteria

The target population comprised adults aged 30–74 years, consistent with the age range for which the BMI-based Framingham equation used in this study was developed to estimate cardiovascular risk and derive cardiovascular age [11]. The analytic sample was drawn from participants in the ENDES adult health module (2014–2024) with complete information and valid measurements for computing the outcome and the stratification variables.

We included participants who simultaneously met the following criteria: (i) availability of at least two valid systolic blood pressure measurements to compute an average value; (ii) valid weight and height measurements to compute body mass index (BMI); (iii) complete information for the variables required by the equation (age, sex, smoking, diabetes, and antihypertensive treatment); and (iv) valid data for the socioeconomic and geographic stratification variables used in the inequality analyses.

We excluded records with biologically implausible values or values compatible with measurement/data-entry error in key components (systolic blood pressure <50 or >300 mmHg; body mass index <10 or >80 kg/m^2^), as well as those with invalid sampling weights (≤0) or missing data in any variables essential for the analysis (complete-case analysis).

### 2.4. Study Variables

#### 2.4.1. Outcome

The primary outcome was predicted excess cardiovascular age, defined as the arithmetic difference between estimated cardiovascular age and the participant’s chronological age. Positive values indicate that the risk-equation-derived cardiovascular age is greater than chronological age, whereas negative values indicate a predicted cardiovascular age lower than chronological age. This metric should be interpreted as an estimated risk-factor burden, not as observed cardiovascular disease or objective subclinical cardiovascular damage.

Cardiovascular age was estimated using the BMI-based non-laboratory Framingham equation described by D’Agostino and colleagues [11], suitable for settings where population lipid profiles are unavailable. Operationally, the calculation followed two steps: (i) estimating the 10-year absolute risk of cardiovascular disease using the sex-specific model coefficients (including antihypertensive treatment as a modifier of the blood pressure component); and (ii) deriving cardiovascular age by solving for the age that yields the same individual risk under an ideal risk-factor profile: systolic blood pressure (SBP) 125 mmHg, BMI 22.5 kg/m^2^, non-smoker, no diabetes, and no antihypertensive treatment. To preserve interpretability and reduce the influence of extreme values, cardiovascular age was operationally restricted to 30–100 years.

#### 2.4.2. Main Exposure Variables

Socioeconomic position was operationalized using the household wealth index provided by INEI, constructed through principal components analysis of household assets, housing conditions, and access to basic services. We used the classification into wealth quintiles (Q1–Q5), from the poorest (Q1) to the richest (Q5). For inequality analyses, this indicator was treated as an ordinal variable representing the socioeconomic gradient.

Geographic variables included the natural region of residence, categorized as Metropolitan Lima (Province of Lima and the Constitutional Province of Callao), rest of the Coast, Highlands, and Amazon; the area of residence (urban/rural) according to INEI classification; and the department of residence, considering the 25 administrative units (including Callao).

As an additional socioeconomic indicator, educational attainment was included as the highest level achieved, classified as: none/early childhood, primary, secondary, and higher (technical or university; complete or incomplete).

#### 2.4.3. Covariates and Components of the Framingham Equation

Stratification variables used for descriptive purposes included sex (women/men), chronological age in years (used continuously in the equation and categorized for presentation as 30–39, 40–49, 50–59, and 60–74 years), and survey year (2014–2024).

The components of the Framingham equation were operationalized as follows. Systolic blood pressure was calculated as the arithmetic mean of the two measurements taken during the household visit. BMI was calculated as weight in kilograms divided by height in meters squared. Current smoking was defined as self-reported cigarette smoking in the last 30 days. Diabetes was defined by self-reported prior medical diagnosis of diabetes mellitus. Antihypertensive treatment was defined as self-reported current use of medications to control blood pressure.

### 2.5. Statistical Analysis

All analyses incorporated the complex sampling design of ENDES by specifying primary sampling units (clusters/PSUs), strata, and sampling weights (expansion factors). For the combined 2014–2024 period, sampling weights were re-scaled by dividing by the number of included rounds (k = 11) so that the weighted total approximately represents the average annual population during the period and avoids interpreting the sum of weights as an “accumulated population” over 11 years. In year-stratified analyses, we used the original weights corresponding to each annual round.

Population characteristics were described using unweighted absolute frequencies (n) and weighted percentages with 95% confidence intervals (CI) for categorical variables. For continuous variables, we estimated weighted means with standard deviations (SD). Estimates were presented for the total population and stratified by sex.

Excess cardiovascular age was described using weighted means with 95% CIs across socioeconomic and geographic categories. Additionally, we estimated the proportion of participants with predicted excess cardiovascular age ≥ 10 years as a descriptive threshold for substantial predicted excess; this threshold was not interpreted as an observed clinical outcome.

Socioeconomic inequality in excess cardiovascular age was quantified using a set of complementary indicators that capture both absolute and relative differences along the wealth gradient. First, we estimated gaps between extreme quintiles: the absolute gap as the difference in weighted means between the richest (Q5) and poorest (Q1) quintiles (Q5–Q1), and the relative gap as the ratio of these weighted means (Q5/Q1). These measures directly describe the magnitude and direction of inequality between the most socioeconomically distant groups.

We also calculated the Slope Index of Inequality (SII) using survey-weighted linear regression, modeling excess cardiovascular age as a function of the wealth ridit score, which represents each individual’s cumulative relative position in the socioeconomic distribution. The ridit was obtained by assigning each quintile the midpoint of its weighted cumulative proportion, defined as the weighted cumulative proportion of lower categories plus 0.5 times the proportion of the corresponding category. The regression coefficient represents the expected absolute difference in excess cardiovascular age between the theoretical extremes of the gradient (ridit = 0 vs. ridit = 1) and was reported with its corresponding 95% confidence interval (95% CI).

The Relative Index of Inequality (RII) was derived from the same model used for the SII and was calculated as the ratio between the predicted excess cardiovascular age at the ridit extremes (prediction at ridit = 1 divided by the prediction at ridit = 0). Because excess cardiovascular age can take negative values at the individual level, the RII was interpreted as a relative measure based on predicted means, and its applicability depends on positive predictions at both extremes.

Finally, we estimated the concentration index (CI) and constructed the corresponding concentration curve. The CI was calculated using the covariance method, defined as CI = (2/μ)·cov(y,r), where μ is the weighted mean excess cardiovascular age, y is the individual value of excess, and r is the individual’s weighted fractional rank in the wealth distribution. Positive CI values indicate concentration of excess among higher-wealth strata (pro-rich pattern), whereas negative values indicate concentration among lower-wealth strata (pro-poor pattern). The concentration curve was generated by plotting the cumulative proportion of excess (y-axis) against the cumulative proportion of the population ranked by wealth (x-axis). All socioeconomic indicators were estimated for the total population, and analyses were additionally stratified by sex.

To further explore the reverse socioeconomic gradient, we conducted exploratory survey-weighted interaction analyses between the wealth ridit score and sex, area of residence, and age group. We also estimated stratum-specific SIIs using wealth ridit scores recalculated within each stratum. Finally, to characterize the risk-factor pattern underlying the gradient, we described the components of the Framingham-based cardiovascular age equation across wealth quintiles. These analyses were descriptive and were not interpreted as causal mediation.

Geographic variation in excess cardiovascular age was examined by comparing weighted means and their corresponding 95% CIs across territorial units, including natural region, area of residence (urban/rural), and department. To facilitate interpretation of spatial inequalities, differences between the regions with the highest and lowest excess were summarized in both absolute terms (difference in means) and relative terms (ratio of means), quantifying the magnitude of the geographic gradient.

Department-level estimates were displayed using complementary graphical approaches. First, choropleth maps of mean excess by department were created to identify spatial patterns and potential regional clustering. Second, caterpillar plots were constructed, ordering departmental estimates from lowest to highest along with their 95% CIs and using the national mean as a reference to facilitate visual comparison between departments and the country average. For visual orientation, the choropleth map includes schematic labels for the broad geographic domains used in the analysis and an arrow identifying Metropolitan Lima/Callao.

We estimated the weighted mean excess cardiovascular age for each year from 2014 to 2024 (overall and by sex) and graphically presented the estimates with 95% CIs. Given the descriptive nature of the study, we did not perform formal trend tests, interpreting temporal patterns exploratorily while considering possible contextual changes (e.g., the COVID-19 pandemic) and operational modifications to the survey.

Analyses were conducted in Stata v17.0 (StataCorp LLC, College Station, TX, USA) using survey commands (svy). Visualizations and maps were produced in R v4.3 (R Foundation for Statistical Computing, Vienna, Austria) using the sf package for spatial data handling and ggplot2 for visualization. The choropleth map was constructed using geom_sf() for departmental geometries, scale_fill_gradientn() for the color scale, geom_sf_text() for department-level labels, and theme_minimal() for graphical formatting. Department-level geometries were obtained from the GADM Database of Global Administrative Areas, version 4.1, using the Peru level-1 administrative boundary file [12].

### 2.6. Ethical Considerations

This study used only secondary, publicly available, fully anonymized data from the Peruvian National Institute of Statistics and Informatics (INEI) microdata repository. ENDES obtains verbal informed consent from all participants before each interview and measurement. Public data contain no personal identifiers that allow direct or indirect participant identification. Additionally, in accordance with Research Ethics Committee guidelines for secondary data use, studies using public, anonymized databases do not require additional ethics approval or renewed informed consent from participants [13]. Nevertheless, we adhered to ethical principles of confidentiality and responsible data use, reporting only aggregated results that do not permit identification of specific individuals.

## 3. Results

### 3.1. Participant Selection

From ENDES 2014–2024 (adult health module), we initially had 260,574 interviewed individuals across 11 annual rounds. After restricting to the 30–74-year age range, 165,109 participants were included. We then excluded several observations, yielding a final analytic sample of 163,889 adults (Supplementary Material Figure S1). Overall, exclusions due to quality/missingness after age restriction represented 0.74% of the age-eligible population.

### 3.2. Characteristics of the Study Population

The analytic sample included 163,889 adults aged 30–74 years. Estimates are presented as unweighted sample sizes (n) and weighted percentages/means that account for the complex survey design. Women accounted for 50.6% of the population (n = 86,914), and mean age was 47.8 years (SD: 11.9). By age group, 30.6% were 30–39 years, 27.9% were 40–49, 23.3% were 50–59, and 18.2% were 60–74 years.

Socioeconomically, 18.2% belonged to the poorest wealth quintile (Q1) and 22.0% to the richest quintile (Q5). Regarding education, 35.5% reported secondary education and 27.1% higher education. Geographically, 32.5% lived in Metropolitan Lima, 24.3% in the rest of the Coast, 27.8% in the Highlands, and 15.4% in the Amazon; additionally, 76.8% lived in urban areas.

Regarding risk factors, mean systolic blood pressure was 121.4 mmHg (SD: 17.8), higher in men (126.1 mmHg) than in women (117.2 mmHg). Mean BMI was 27.4 kg/m^2^ (SD: 4.8), with higher values in women (27.9 kg/m^2^) than in men (26.8 kg/m^2^). The prevalence of current smoking was 7.5%, with a marked sex difference (women: 2.8%; men: 12.8%). The prevalence of self-reported diabetes was 6.0%, and 11.3% reported antihypertensive treatment (Table 1). Finally, the mean 10-year cardiovascular risk was 8.2% (SD: 9.4), higher in men (11.7%) than in women (5.1%) (Table 1).

### 3.3. Temporal Variation in Excess Cardiovascular Age (2014–2024)

**Figure 1:**
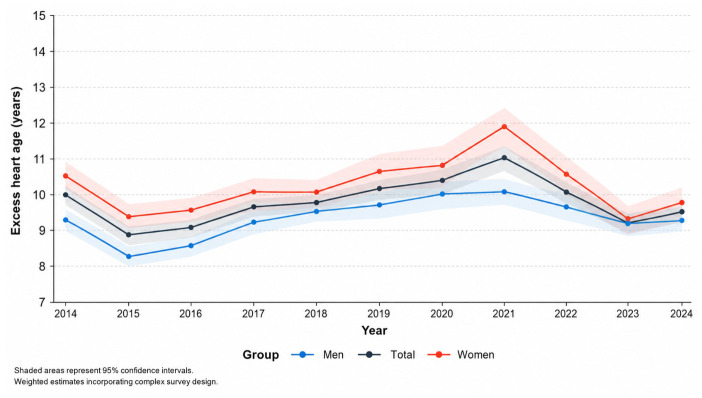
Temporal trends in predicted excess cardiovascular age, 2014–2024.

Temporal variation in excess cardiovascular age was observed across 2014–2024. In 2014, the weighted mean was 9.92 years (95% CI: 9.64–10.20). Excess then decreased in 2015 (8.76 years) and subsequently increased, reaching a peak in 2021 (10.91 years; 95% CI: 10.57–11.25). In later years, estimates declined, with 9.17 years in 2023 and 9.45 years in 2024—values close to the period mean. When stratified by sex, the increase in 2021 was more pronounced in women (11.82 years; 95% CI: 11.26–12.38), whereas in men the highest value was also observed in 2021 (10.02 years) (Figure 1).

### 3.4. Distribution of Excess Cardiovascular Age in the Population

Among Peruvian adults aged 30–74 years, mean predicted excess cardiovascular age was 9.64 years (95% CI: 9.48–9.80), indicating that, according to the risk equation, predicted cardiovascular age exceeded chronological age by approximately a decade on average. Estimates were similar by sex: 9.73 years in women (95% CI: 9.52–9.94) and 9.54 years in men (95% CI: 9.33–9.75) (Table 2).

Excess cardiovascular age increased progressively with chronological age, from 5.12 years in the 30–39-year group to 14.56 years in the 60–74-year group. In sex-stratified analyses, excess was higher in women in the 30–59-year groups, whereas in the 60–74-year group it was higher in men (Table 2).

### 3.5. Socioeconomic Inequalities in Excess Cardiovascular Age

A pro-rich socioeconomic gradient was observed in excess cardiovascular age, with a progressive increase across wealth quintiles. The weighted mean increased from 7.14 years (95% CI: 7.00–7.29) in the poorest quintile (Q1) to 11.25 years (95% CI: 11.01–11.50) in the richest quintile (Q5). The absolute gap between extreme quintiles (Q5–Q1) was 4.11 years (95% CI: 3.82–4.40), and the relative gap (Q5/Q1) was 1.58 (Table 3).

**Figure 2:**
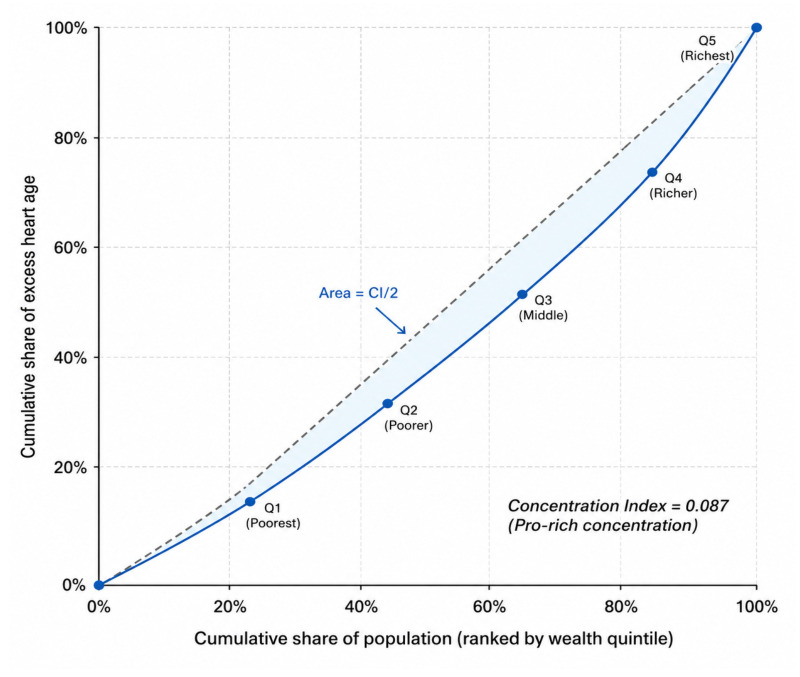
Concentration curve for predicted excess cardiovascular age.

When considering the full gradient using standardized indicators, the SII was 5.04 years (95% CI: 4.71–5.37), indicating an absolute increase of about five years in predicted excess cardiovascular age from the poorest to the richest end of the socioeconomic gradient. The RII was 1.71, and the concentration index was 0.087, consistent with concentration of predicted excess among higher-wealth strata (Table 3). Accordingly, the concentration curve lay below the line of equality (Figure 2), reflecting that predicted excess cardiovascular age was concentrated in groups with higher socioeconomic position.

A similar pattern was observed when stratifying by education: mean excess increased from 8.23 years in participants with no/early education to 10.89 years among those reporting higher education (Table 2), reinforcing the consistency of the socioeconomic gradient.

The gradient was more pronounced in men than in women. In men, the SII reached 6.14 years (95% CI: 5.80–6.48), the RII was 1.99, and the concentration index was 0.111; in women, the SII was 3.90 years (95% CI: 3.36–4.44), the RII was 1.48, and the concentration index was 0.065, indicating a less intense pro-rich concentration among women (Table 3; Supplementary Material Figure S2).

Exploratory survey-weighted interaction analyses (Supplementary Material Table S2) showed that the pro-rich gradient in predicted excess cardiovascular age varied across major population strata. The gradient was steeper among men than women: 3.90 years in women versus 6.14 years in men, with an interaction difference of 2.24 years (95% CI: 1.63–2.86). The gradient also increased progressively across age groups, from 1.21 years among adults aged 30–39 years to 9.71 years among those aged 60–74 years. Analyses by area of residence suggested that interpretation depended on how socioeconomic position was ranked: using the national wealth ridit, the gradient was larger in rural than urban areas, whereas residence-specific SIIs based on within-residence wealth ranks were smaller and broadly similar. Component-specific analyses showed that higher wealth quintiles had higher BMI, systolic blood pressure, self-reported diabetes, antihypertensive treatment, and current smoking prevalence, supporting that the pro-rich gradient reflected clustering of several risk-factor components rather than a single isolated factor.

### 3.6. Geographic Inequalities in Excess Cardiovascular Age

Geographic heterogeneity in excess cardiovascular age was evident at multiple scales. At the macro-regional level, residents of Metropolitan Lima had the highest excess (11.17 years; 95% CI: 10.95–11.39) and residents of the Highlands the lowest (7.45 years; 95% CI: 7.33–7.57), with an absolute Lima–Highlands gap of 3.72 years and a relative gap of 1.50 (Table 3). Urban residence was also associated with substantially higher excess (10.28 years; 95% CI: 10.17–10.40) than rural residence (7.25 years; 95% CI: 7.10–7.39), with an urban–rural gap of 3.04 years (ratio 1.42). This pattern held in both sexes, although the absolute magnitude for the urban–rural comparison was larger in men (Table 2 and Table 3).

**Figure 3:**
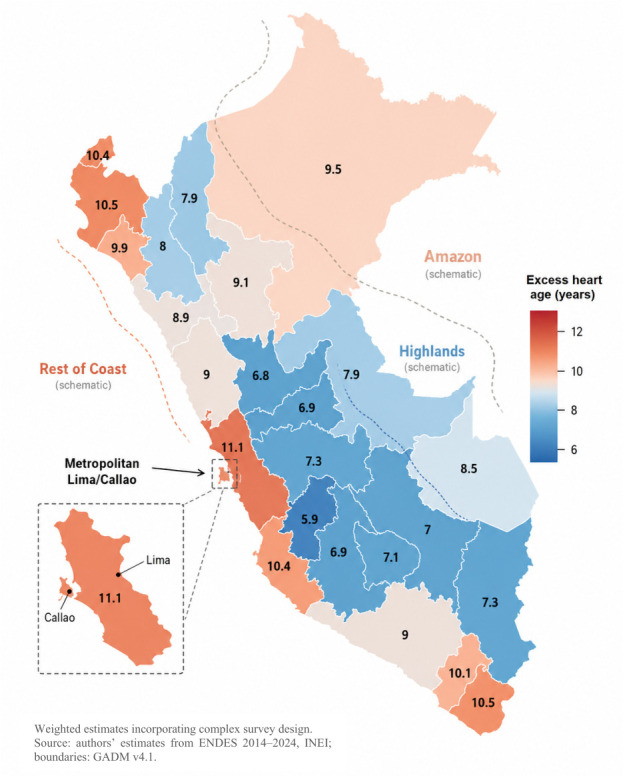
Geographic distribution of predicted excess cardiovascular age by department. Dashed lines and labels provide a schematic orientation to the broad geographic domains used in the analysis—Metropolitan Lima/Callao, Rest of Coast, Highlands, and Amazon—and should not be interpreted as exact ecological boundaries. The arrow identifies Metropolitan Lima/Callao. Source: authors’ own elaboration based on ENDES 2014–2024 microdata from the Instituto Nacional de Estadística e Informática (INEI) [10]; departmental geometries were obtained from the GADM Database of Global Administrative Areas, version 4.1 [12].

At the departmental level, predicted excess cardiovascular age spanned nearly six years between extremes. Huancavelica, a predominantly Andean highland department in south-central Peru, showed the lowest excess (5.93 years; 95% CI: 5.56–6.30). In contrast, Callao, the coastal Constitutional Province adjacent to Metropolitan Lima and part of the country’s largest urban conurbation, showed the highest excess (11.83 years; 95% CI: 11.47–12.19). Lower values were predominantly observed in Andean departments, whereas higher values clustered in coastal departments and metropolitan areas (Figure 3).

Departmental estimates are presented in the caterpillar plot (Supplementary Material Figure S5), using the national mean (9.64 years) as a reference. For analytical transparency, departments were classified as above or below the national mean according to the defined criterion (e.g., 95% CI not including the national mean).

## 4. Discussion

### 4.1. Main Findings

In this repeated cross-sectional series based on ENDES 2014–2024 (n = 163,889; 30–74 years), cardiovascular age was estimated using the BMI-based non-laboratory Framingham score, and excess was defined as the difference between cardiovascular age and chronological age [11]. Mean predicted excess was 9.64 years, indicating that the joint risk-factor profile (blood pressure, diabetes, smoking, BMI, and antihypertensive treatment) translates, at the population level, into a cardiovascular age roughly a decade above chronological age. This value should be interpreted as a risk-equation-derived summary of risk-factor burden, rather than as observed cardiovascular disease or objective subclinical vascular damage.

From an equity perspective, the study documents a consistent pro-rich concentration pattern in predicted excess cardiovascular age, supported by absolute and relative gaps between extreme quintiles and by summary indicators (SII, RII, and concentration index), with a steeper gradient in men. Substantial geographic inequalities were also observed: excess was higher in Metropolitan Lima and the Coast, as well as in urban areas, with marked heterogeneity across departments. Taken together, these results show that estimated cardiovascular risk, integrated into an age-based metric, is not homogeneously distributed in the country and that social and territorial inequalities in risk-factor burden coexist.

The sex- and age-specific patterns should be interpreted cautiously because predicted cardiovascular age is a sex-specific, risk-factor–equivalent metric, not a direct measure of cardiovascular events. In our data, women had higher excess cardiovascular age than men in the 30–59-year age groups, whereas men had higher excess in the 60–74-year group. This apparent reversal does not necessarily contradict known sex differences in clinical cardiovascular disease, because the metric combines different components whose distributions vary by sex and age. For example, women had higher mean BMI and a higher prevalence of antihypertensive treatment, whereas men had higher mean systolic blood pressure and substantially higher smoking prevalence. Thus, the observed pattern most likely reflects heterogeneity in the composition of risk-factor profiles across sex and age strata rather than a claim that clinical cardiovascular disease incidence is higher in younger women than in men.

The exploratory interaction and component analyses strengthen the interpretation of the reverse socioeconomic gradient without converting the study into a causal decomposition. The gradient was not driven by a single score component; rather, higher-wealth strata showed broader clustering of risk-factor components. At the same time, self-reported diabetes and antihypertensive treatment may be affected by differential diagnosis and access to care, so part of the pro-rich gradient could reflect detection bias rather than true biological burden alone.

### 4.2. Comparison with Other Studies

The magnitude of excess observed in Peru (9.64 years) is comparable to recent estimates in other populations using the Framingham-derived “heart age” approach. In the United States, an analysis of NHANES 2015–March 2020 reported a mean excess of 8.6 years in men and 5.9 years in women [14]. In that context, the Peruvian mean excess is similar to that described for U.S. men and higher than that estimated for women, which may reflect population differences in cardiometabolic risk-factor distributions and in the history of implementing preventive strategies.

At a multinational scale, an analysis of 41 WHO STEPS surveys showed that heart age is generally higher than chronological age in low- and middle-income countries, with substantial regional heterogeneity. In that study, abdominal obesity, smoking, and diabetes were associated with a higher probability of excess; in contrast, higher education and employment were inversely associated with excess [7]. This finding contrasts with the pro-rich gradient observed in Peru (by wealth and education) and suggests that the direction of inequalities in risk-factor–based metrics is not uniform across low- and middle-income countries, and likely depends on the stage and speed of the nutritional transition, urbanization patterns, and the organization of prevention and care systems.

The pro-rich pattern identified should be interpreted recognizing that inequalities differ when examining risk factors versus events/mortality. Global reviews have documented that, in most countries, socioeconomically disadvantaged groups tend to have higher risk of dying from non-communicable diseases, although the gradient depends on the stage of economic development and on social and health policies [15]. In the PURE cohort (20 countries), major cardiovascular events were more frequent among people with lower education across all income levels; however, risk-factor profiles showed divergent patterns: in high-income countries, risk factors decreased with higher education, whereas in low-income countries they increased with education, highlighting the role of inequalities in prevention, timely diagnosis, and treatment in shaping the ultimate distribution of outcomes [3]. This framework is consistent with the possibility that, in transitional contexts, excess cardiovascular age (a risk-factor–based metric) may initially concentrate in more advantaged strata, even as the burden of events shifts toward less advantaged groups due to differences in access and disease control.

In middle-income countries, positive (pro-rich) gradients have been described for cardiometabolic risk factors. In India, for example, markedly positive socioeconomic gradients were observed for obesity, diabetes, and hypertension, and a high proportion of the population burden was concentrated in higher socioeconomic groups [16]. In Latin America and the Caribbean, an analysis of national surveys (1998–2017) showed a sustained rise in obesity with changing patterns by wealth, education, and area of residence; in the most recent surveys, the largest wealth difference among men was observed in Peru, and obesity was consistently higher in urban than rural areas in most countries [4]. This regional evidence aligns with our findings by suggesting that predicted cardiovascular age summarizes socioeconomic clustering of cardiometabolic risk-factor components in urban and higher-wealth settings, rather than demonstrating a causal process of risk accumulation.

Finally, the geographic inequalities observed (higher excess on the Coast/Metropolitan Lima and lower in the Highlands) are consistent with prior literature. In 38 low- and middle-income countries, urban–rural differences in BMI were substantially attenuated after adjusting for socioeconomic indicators, suggesting that part of the “urban effect” reflects the concentration of wealth and obesogenic lifestyles in cities [17]. In Peru, the PERU MIGRANT study showed a rural–migrant–urban gradient for obesity and diabetes, supporting that cumulative exposure to urban environments is associated with less favorable cardiometabolic profiles [18]. In terms of potential impact, an integrated metric of modifiable risk factors is particularly relevant because INTERHEART showed that a substantial proportion of myocardial infarction risk is explained by a relatively limited set of modifiable factors present across regions, reinforcing the value of interventions targeting blood pressure, smoking, and metabolism [19].

### 4.3. Surveillance-Oriented Implications for Cardiovascular Risk-Factor Prevention

From a surveillance perspective, the main implication of these findings is not that a specific intervention was tested, but that the estimated burden of modifiable cardiovascular risk factors is socially and territorially patterned. These results may inform prevention strategies in Peru by supporting universal cardiovascular risk-factor control with proportionate intensification in territories where predicted excess cardiovascular age is currently higher, particularly urban, coastal, and metropolitan settings. At the same time, lower estimated excess in rural and highland areas should not be interpreted as absence of need, because underdiagnosis and reduced access to preventive care may attenuate measured risk-factor burden.

Predicted cardiovascular age may also be useful as a risk-communication format within structured primary-care prevention, especially when linked to blood pressure control, smoking cessation, diabetes detection, and weight management [6,8]. However, it should complement—not replace—locally calibrated risk assessment and outcome-based surveillance. Evidence-based policy domains such as hypertension control, tobacco control, and healthier food environments are consistent with the risk factors summarized by this metric [20,21,22,23,24,25], but their effectiveness was not evaluated in the present analysis. Similarly, the 2021 peak should be interpreted descriptively, not as evidence of pandemic-related service disruption or post-COVID cardiovascular outcomes, which were outside the scope of this study [26,27].

The transferable lesson for other middle-income countries is therefore methodological rather than numerical: countries with marked socioeconomic, urban–rural, and regional heterogeneity should measure the local direction and magnitude of risk-factor inequalities instead of assuming that gradients observed elsewhere apply uniformly.

### 4.4. Limitations

This study has limitations inherent to its repeated cross-sectional design. ENDES does not follow the same individuals over time; therefore, the temporal patterns described here cannot be interpreted as individual trajectories, causal accumulation of exposure, or transitions in cardiovascular risk at the person level. Similarly, associations by wealth, residence, and department are descriptive and should not be interpreted causally. Predicted excess cardiovascular age was derived from the BMI-based non-laboratory Framingham equation [11] and therefore reflects estimated risk-factor burden rather than observed cardiovascular events or objective measures of subclinical cardiovascular disease. To our knowledge, this specific heart-age formulation has not been externally validated or recalibrated against cardiovascular outcomes in Peru. Consequently, the absolute level of predicted excess cardiovascular age may be affected by miscalibration; internal comparisons may be more robust because the same algorithm was applied to all participants, but differential miscalibration by sex, socioeconomic position, or region could still influence the magnitude of inequality estimates.

Several components of the equation were self-reported, including smoking, prior diabetes diagnosis, and antihypertensive treatment. These variables are susceptible to information bias and differential underdiagnosis. In particular, participants with greater socioeconomic resources or better access to health services may be more likely to report diagnosed diabetes or antihypertensive treatment, potentially inflating the apparent pro-rich gradient in predicted cardiovascular age; conversely, undiagnosed disease in poorer or remote populations may lead to underestimation of risk-factor burden. The wealth index is asset-based and does not capture all dimensions of socioeconomic position, and the analysis did not include lipid biomarkers, diet, physical activity, health care access, or longitudinal clinical outcomes. Finally, although ENDES is nationally and departmentally representative, estimates for smaller departments may have lower precision.

## 5. Conclusions

Among Peruvian adults aged 30–74 years, mean predicted excess cardiovascular age was close to a decade, and a substantial proportion had predicted excess cardiovascular age ≥ 10 years. A consistent pro-rich inequality pattern and marked geographic heterogeneity were documented, with higher values in urban areas and in Metropolitan Lima/the Coast. Because this metric is derived from a non-laboratory risk equation, the results should be interpreted as inequalities in estimated risk-factor burden, not as evidence of observed cardiovascular disease, subclinical cardiovascular damage, causal mechanisms, or tested intervention effects.

These findings may inform cardiovascular risk-factor surveillance and primary-care prevention with a territorial and equity perspective. Rather than identifying tested interventions, the results indicate where intensified detection and management of modifiable risk factors may be most relevant. Similar surveillance-oriented approaches may be useful for other middle-income countries with strong geographic and socioeconomic heterogeneity, but the direction and magnitude of inequality should be locally assessed rather than extrapolated from Peru.

## Figures and Tables

**Table 1:** Characteristics of the study population by sex, ENDES 2014–2024.

Characteristic	Total (n = 163,889)	Women (n = 86,914)	Men (n = 76,975)
Age, years, mean (SD)	47.8 (11.9)	47.6 (11.8)	48.0 (12.0)
Age group, n (%)			
30–39 years	50,124 (30.6)	27,033 (31.1)	23,091 (29.8)
40–49 years	45,678 (27.9)	24,512 (28.2)	21,166 (27.5)
50–59 years	38,234 (23.3)	20,145 (23.2)	18,089 (23.5)
60–74 years	29,853 (18.2)	15,224 (17.5)	14,629 (19.0)
Educational level, n (%)			
No schooling/Pre-primary	8956 (5.5)	7234 (8.3)	1722 (2.2)
Primary	52,345 (31.9)	31,456 (36.2)	20,889 (27.1)
Secondary	58,234 (35.5)	28,567 (32.9)	29,667 (38.5)
Higher	44,354 (27.1)	19,657 (22.6)	24,697 (32.1)
Wealth quintile, n (%)			
Q1 (Poorest)	46,897 (18.2)	25,123 (18.5)	21,774 (17.9)
Q2 (Poor)	39,104 (18.8)	20,856 (19.1)	18,248 (18.6)
Q3 (Middle)	31,409 (20.0)	16,534 (19.8)	14,875 (20.1)
Q4 (Rich)	25,794 (21.0)	13,567 (20.8)	12,227 (21.2)
Q5 (Richest)	20,685 (22.0)	10,834 (21.8)	9851 (22.2)
Natural region, n (%)			
Metropolitan Lima	17,234 (32.5)	9123 (32.8)	8111 (32.2)
Rest of Coast	38,567 (24.3)	20,345 (24.1)	18,222 (24.5)
Highlands	72,456 (27.8)	38,234 (27.9)	34,222 (27.7)
Amazon	35,632 (15.4)	19,212 (15.2)	16,420 (15.6)
Area of residence, n (%)			
Urban	94,567 (76.8)	50,234 (77.1)	44,333 (76.5)
Rural	69,322 (23.2)	36,680 (22.9)	32,642 (23.5)
Cardiovascular risk factors			
SBP, mmHg, mean (SD)	121.4 (17.8)	117.2 (17.1)	126.1 (17.5)
BMI, kg/m^2^, mean (SD)	27.4 (4.8)	27.9 (5.1)	26.8 (4.3)
Current smoker, n (%)	12,345 (7.5)	2456 (2.8)	9889 (12.8)
Diabetes, n (%)	9876 (6.0)	5234 (6.0)	4642 (6.0)
Antihypertensive treatment, n (%)	18,567 (11.3)	11,234 (12.9)	7333 (9.5)
Cardiovascular indicators			
Cardiovascular age, years, mean (SD)	57.4 (15.2)	57.3 (14.8)	57.5 (15.6)
Excess cardiovascular age, years, mean (SD)	9.64 (11.2)	9.73 (10.8)	9.54 (11.6)
10-year CVD risk, %, mean (SD)	8.2 (9.4)	5.1 (6.2)	11.7 (11.2)

Abbreviations: SD, standard deviation; SBP, systolic blood pressure; BMI, body mass index; Q1–Q5, wealth quintiles (Q1 = poorest, Q5 = richest). n values correspond to unweighted absolute frequencies; percentages and means are weighted estimates that account for the complex survey design.

**Table 2:** Predicted excess cardiovascular age by sociodemographic characteristics and sex.

Characteristic	Total	Women	Men
Total	9.64 (9.48; 9.80)	9.73 (9.52; 9.94)	9.54 (9.33; 9.75)
Age group			
30–39 years	5.12 (4.92; 5.32)	5.84 (5.58; 6.10)	4.32 (4.08; 4.56)
40–49 years	8.45 (8.21; 8.69)	9.12 (8.82; 9.42)	7.71 (7.41; 8.01)
50–59 years	12.34 (12.02; 12.66)	12.89 (12.49; 13.29)	11.73 (11.33; 12.13)
60–74 years	14.56 (14.18; 14.94)	13.21 (12.75; 13.67)	15.97 (15.47; 16.47)
Wealth quintile			
Q1 (Poorest)	7.14 (7.00; 7.29)	8.09 (7.83; 8.34)	6.19 (6.03; 6.34)
Q2 (Poor)	8.70 (8.53; 8.87)	9.30 (9.02; 9.58)	8.18 (7.98; 8.38)
Q3 (Middle)	9.88 (9.68; 10.08)	10.32 (10.02; 10.62)	9.43 (9.18; 9.68)
Q4 (Rich)	10.74 (10.52; 10.97)	10.89 (10.57; 11.21)	10.54 (10.24; 10.84)
Q5 (Richest)	11.25 (11.01; 11.50)	11.20 (10.86; 11.54)	11.32 (11.00; 11.64)
Natural region			
Metropolitan Lima	11.17 (10.95; 11.39)	11.54 (11.22; 11.86)	10.78 (10.48; 11.08)
Rest of Coast	10.30 (10.15; 10.46)	10.68 (10.42; 10.94)	9.94 (9.72; 10.16)
Highlands	7.45 (7.33; 7.57)	7.89 (7.69; 8.09)	6.94 (6.78; 7.10)
Amazon	8.32 (8.16; 8.49)	8.72 (8.48; 8.96)	7.89 (7.67; 8.11)
Area of residence			
Urban	10.28 (10.17; 10.40)	10.56 (10.40; 10.72)	9.99 (9.83; 10.15)
Rural	7.25 (7.10; 7.39)	8.11 (7.88; 8.34)	6.36 (6.18; 6.54)
Educational level			
No schooling/Pre-primary	8.23 (7.89; 8.57)	8.45 (8.05; 8.85)	7.56 (6.98; 8.14)
Primary	8.67 (8.49; 8.85)	9.12 (8.86; 9.38)	8.01 (7.77; 8.25)
Secondary	9.45 (9.27; 9.63)	9.78 (9.52; 10.04)	9.13 (8.89; 9.37)
Higher	10.89 (10.67; 11.11)	10.98 (10.66; 11.30)	10.78 (10.48; 11.08)

Values are weighted means with 95% confidence intervals in parentheses. Predicted excess cardiovascular age is defined as the difference between cardiovascular age (calculated using the BMI-based Framingham equation) and chronological age.

**Table 3:** Indicators of socioeconomic inequality in predicted excess cardiovascular age.

Indicator	Total	Women	Men
Gaps by wealth quintile			
Excess in Q1 (poorest), years	7.14	8.09	6.19
Excess in Q5 (richest), years	11.25	11.20	11.32
Absolute gap (Q5–Q1), years	4.11	3.11	5.13
95% CI	(3.82; 4.40)	(1.78; 4.44)	(3.78; 6.48)
Relative gap (Q5/Q1)	1.58	1.38	1.83
Inequality indices			
SII, years	5.04	3.90	6.14
95% CI	(4.71; 5.37)	(3.36; 4.44)	(5.80; 6.48)
RII	1.71	1.48	1.99
Concentration index	0.087	0.065	0.111
Gaps by area of residence			
Absolute gap (Urban–Rural), years	3.04	2.45	3.63
Relative gap (Urban/Rural)	1.42	1.30	1.57
Gaps by natural region			
Absolute gap (Lima–Highlands), years	3.72	3.65	3.84
Relative gap (Lima/Highlands)	1.50	1.46	1.55

Abbreviations: SII, Slope Index of Inequality; RII, Relative Index of Inequality; CI, confidence interval; Q1, poorest quintile; Q5, richest quintile.

## Data Availability

The data presented in this study are available in the INEI microdata repository at https://proyectos.inei.gob.pe/microdatos/ (accessed on 1 March 2026). These data were derived from the following resources available in the public domain: ENDES Peru 2014–2024.

## Supplementary Materials

The following supporting information can be downloaded at https://www.mdpi.com/article/10.3390/jcdd13070318/s1, Table S1: STROBE checklist for cross-sectional studies; Figure S1: Flow diagram of participant selection; Figure S2: Socioeconomic gradient of predicted excess cardiovascular age by sex; Figure S3: Proportion of adults with predicted excess cardiovascular age ≥ 10 years by department; Figure S4: Geographic distribution of predicted excess cardiovascular age by department and sex; Figure S5: Caterpillar plot of predicted excess cardiovascular age by department; Table S2: Exploratory interaction and component analyses of the socioeconomic gradient.
