# Supplementary material for: Predicted Excess Cardiovascular Age and a Reverse Socioeconomic Gradient in a Middle-Income Latin American Country: A Population-Based Analysis of 163,889 Peruvians
Source: J Cardiovasc Dev Dis. 2026 Jul 9;13(7):318. doi: 10.3390/jcdd13070318 (PMC13411265; doi:10.3390/jcdd13070318)

Figure S4. Geographic distribution of excess cardiovascular age by department and sex. Choropleth maps of mean excess cardiovascular age by department, stratified by sex. Panel A: men; Panel B: women. The same color scale was used in both panels to facilitate comparison.

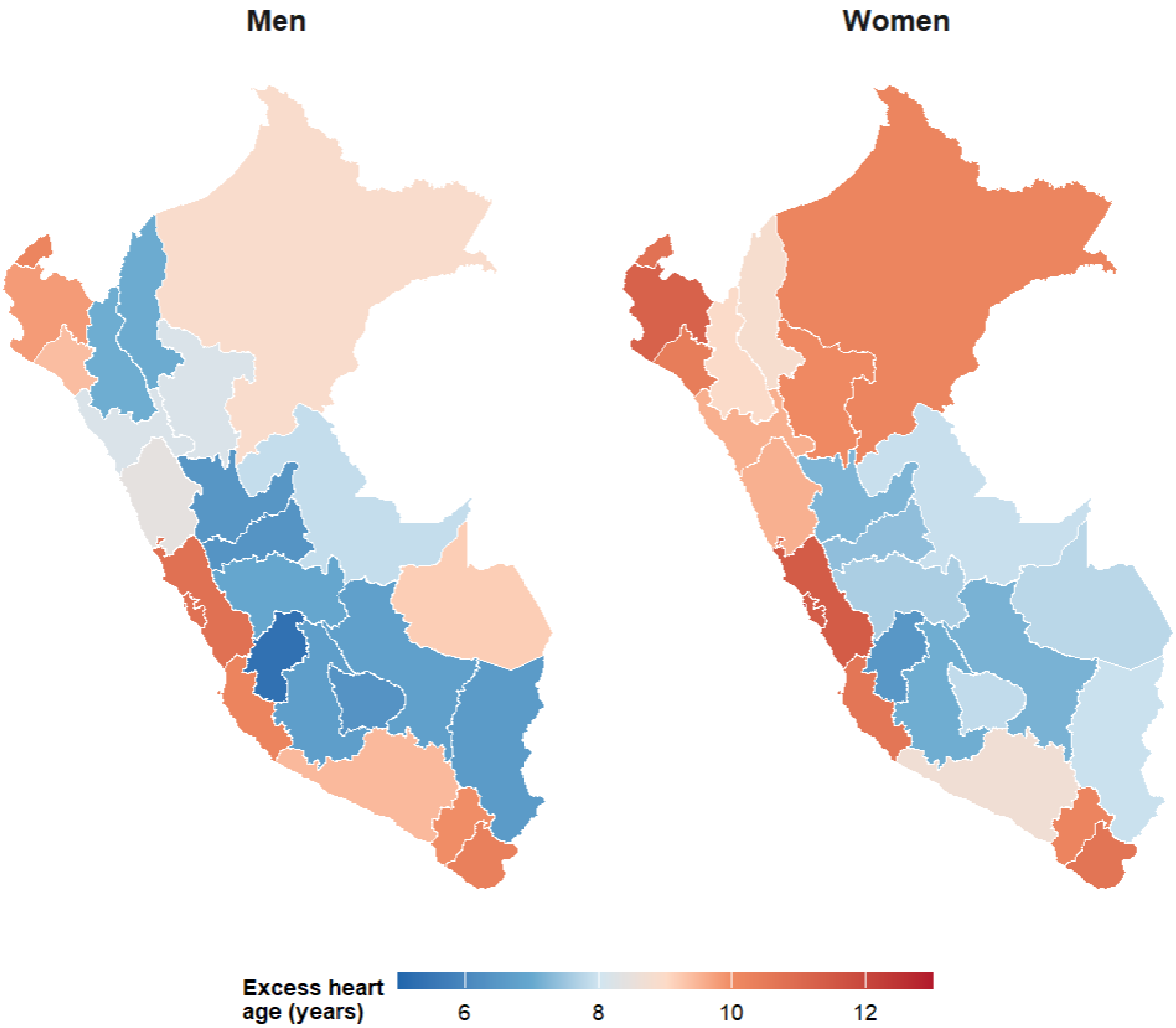

Supplement: Supplementary file 1 [file jcdd-13-00318-s001.zip › Figure S4_Geographic_Distribution_Sex.pdf]
