# Supplementary material for: Predicted Excess Cardiovascular Age and a Reverse Socioeconomic Gradient in a Middle-Income Latin American Country: A Population-Based Analysis of 163,889 Peruvians
Source: J Cardiovasc Dev Dis. 2026 Jul 9;13(7):318. doi: 10.3390/jcdd13070318 (PMC13411265; doi:10.3390/jcdd13070318)
Supplement: Supplementary file 1 [file jcdd-13-00318-s001.zip › Table S1_STROBE.pdf]

Table S1. STROBE (Strengthening the Reporting of Observational Studies in Epidemiology) checklist for cross-sectional studies.

No.	Recommendation	Location in the manuscript
**TITLE AND ABSTRACT**	**TITLE AND ABSTRACT**	**TITLE AND ABSTRACT**
**1**	(a) Indicate the study's design with a commonly used term in the title or the abstract	Abstract, Methods: "Analysis of ENDES Peru 2014–2024". Methods, Study design: observational approach based on repeated cross-sectional surveys.
	(b) Provide in the abstract an informative and balanced summary of what was done and what was found	Abstract: Includes background, objective, methods, results with inequality indices (SII, RII, concentration index), geographic analysis, and conclusions.
**INTRODUCTION**	**INTRODUCTION**	**INTRODUCTION**
**2**	Background/rationale: Explain the scientific background and rationale for the investigation being reported	Introduction, paragraphs 1–4: Global CVD burden, concept of cardiovascular age vs Framingham absolute risk, inequalities in epidemiological transition, knowledge gap in Peru.
**3**	Objectives: State-specific objectives, including any prespecified hypotheses	Introduction, paragraph 4: "The objective of this study was to describe the distribution of excess cardiovascular age and quantify socioeconomic and geographic inequalities."
**METHODS**	**METHODS**	**METHODS**
**4**	Study design: Present key elements of study design early in the paper	Methods, Study design: "Secondary analysis of ENDES 2014–2024 microdata; observational approach based on repeated cross-sectional surveys."
**5**	Setting: Describe the setting, locations, and relevant dates, including periods of recruitment, exposure, follow-up, and data collection	Methods, Data source: ENDES, population-based survey by INEI, two-stage design, period 2014–2024 (11 years).
**6**	Participants: (a) Give the eligibility criteria, and the sources and methods of selection of participants	Methods, Study population, and eligibility criteria: Adults aged 30–74 years, inclusion criteria (valid BP, valid BMI, complete variables), detailed exclusion criteria.
**7**	Variables: Clearly define all outcomes, exposures, predictors, potential confounders, and effect modifiers	Methods, Study variables: Outcome (excess CV age with detailed calculation), exposures (wealth quintile, region, area, education), covariates.
**8**	Data sources/measurement: For each variable of interest, give sources of data and details of methods of assessment (measurement)	Methods, Data source, and Covariates: ENDES includes blood pressure and anthropometry; SBP = average of two measurements; BMI = weight/height^2; smoking, diabetes, and antihypertensive treatment by self-report.

**9**	Bias: Describe any efforts to address potential sources of bias	Methods: Incorporation of the complex sampling design; Limitations: discussion of potential information bias due to self-report.
**10**	Study size: Explain how the study size was arrived at	Methods, Study population: Analytic sample of 163,889 participants derived from ENDES; Supplementary Material 2 (flow diagram).
**11**	Quantitative variables: Explain how quantitative variables were handled in the analyses	Methods, Study variables: CV age restricted to 30–100 years, categorization of chronological age, wealth quintiles via PCA.
**12**	Statistical methods: (a) Describe all statistical methods, including those used to control for confounding	Methods, Statistical analysis: weighted estimates and 95% CIs accounting for the sampling design; absolute/relative gaps; SII via regression on ridit score, RII, and concentration index.
	(b) Describe any methods used to examine subgroups and interactions	Methods, Statistical analysis: Stratification by sex for all inequality indicators.
	(c) Explain how missing data were addressed	Methods, Study population: Exclusion of participants with missing data; Supplementary Material 2 details exclusions.
	(d) If applicable, explain how the sampling strategy was taken into account	Methods, Statistical analysis: svy commands in Stata, specification of clusters, strata, and sampling weights; weights divided by 11 years.
	(e) Describe any sensitivity analyses	Not applicable (descriptive study).
**RESULTS**	**RESULTS**	**RESULTS**
**13**	Participants: (a) Report the number of individuals at each stage of the study	Results: 260,574 initial → 163,889 final; Supplementary Material 2 (flow diagram).
	(b) Give reasons for non-participation at each stage	Supplementary Material 2: exclusions due to age out of range (95,465), invalid SBP (891), invalid BMI (293), invalid weights (36).
	(c) Consider the use of a flow diagram	Supplementary Material 2: Flow diagram of participant selection (STROBE).
**14**	Descriptive data: (a) Give characteristics of study participants and information on exposures and potential confounders	Results, Characteristics of the study population; Table 1: Characteristics by sex (age, education, quintile, region, area, CVD risk factors).
	(b) Indicate the number of participants with missing data for each variable of interest	Results, Participant selection: exclusions for SBP, BMI, and sampling weights; Supplementary Material 2 (flow diagram).

**15**	Outcome data: Report numbers of outcome events or summary measures	Results: Mean 9.64 years (95% CI: 9.48–9.80), 35.8% with excess ≥10 years; Table 2.
**16**	Main results: (a) Give unadjusted estimates and, if applicable, confounder-adjusted estimates and their precision	Results, Inequalities: absolute/relative gaps, SII 5.04 years (95% CI: 4.71–5.37), RII 1.71, concentration index 0.087; Table 3.
	(b) Report category boundaries when continuous variables were categorized	Methods: wealth quintiles (Q1–Q5), age groups (30–39, 40–49, 50–59, 60–74 years).
	(c) If relevant, consider translating estimates of relative risk into absolute risk for a meaningful time period	Not applicable (continuous outcome).
**17**	Other analyses: Report other analyses done—e.g., analyses of subgroups and interactions, and sensitivity analyses	Results: sex-stratified analyses, geographic analysis by department, temporal trends, concentration curve (Figure 2).
**DISCUSSION**	**DISCUSSION**	**DISCUSSION**
**18**	Key results: Summarize key results with reference to study objectives	Discussion, Main findings: Mean excess 9.64 years, reverse (pro-rich) socioeconomic gradient, SII 5.04 years, geographic inequalities.
**19**	Limitations: Discuss limitations of the study, taking into account sources of potential bias or imprecision	Discussion, Limitations: cross-sectional design (no causality), Framingham equation, and potential miscalibration, self-reported variables, and wealth index limitations.
**20**	Interpretation: Give a cautious overall interpretation considering objectives, limitations, multiplicity of analyses, results from similar studies, and other relevant evidence	Discussion, Comparison: NHANES; WHO STEPS surveys (41 countries); PURE; evidence in transitional countries (e.g., India) and studies relevant to Peru/Latin America (obesity in LAC, PERU MIGRANT, INTERHEART).
**21**	Generalisability: Discuss the generalisability (external validity) of the study results	Discussion, Implications: nationally and departmentally representative results; pattern consistent with middle-income countries undergoing epidemiological transition.
**OTHER INFORMATION**	**OTHER INFORMATION**	**OTHER INFORMATION**
**22**	Funding: Give the source of funding and the role of the funders for the present study	Declarations: This study was funded by the Vice-Rectory for Research of the Universidad Nacional Toribio Rodríguez de Mendoza de Amazonas.
