# Supplementary material for: Predicted Excess Cardiovascular Age and a Reverse Socioeconomic Gradient in a Middle-Income Latin American Country: A Population-Based Analysis of 163,889 Peruvians
Source: J Cardiovasc Dev Dis. 2026 Jul 9;13(7):318. doi: 10.3390/jcdd13070318 (PMC13411265; doi:10.3390/jcdd13070318)
Supplement: Supplementary file 1 [file jcdd-13-00318-s001.zip › Table S2_Exploratory_Analyses.pdf]

Supplementary Material. Exploratory interaction and component analyses of the socioeconomic gradient

Table S2. Exploratory analyses of the reverse socioeconomic gradient in predicted excess cardiovascular age.

Panel A. Survey-weighted interaction slopes using the national wealth ridit.

Analysis	Stratum/contrast	Estimate (95% CI)	p value
Wealth ridit × sex	Women: wealth-gradient slope	3.90 (3.36; 4.44)	<0.001
Wealth ridit × sex	Men: wealth-gradient slope	6.14 (5.80; 6.48)	<0.001
Wealth ridit × sex	Interaction: men minus women	2.24 (1.63; 2.86)	<0.001
Wealth ridit × residence	Urban: wealth-gradient slope	3.68 (3.23; 4.13)	<0.001
Wealth ridit × residence	Rural: wealth-gradient slope	5.09 (4.00; 6.19)	<0.001
Wealth ridit × residence	Interaction: rural minus urban	1.41 (0.23; 2.59)	0.019
Wealth ridit × age group	30–39: wealth-gradient slope	1.21 (0.98; 1.44)	<0.001
Wealth ridit × age group	40–49: wealth-gradient slope	2.32 (1.84; 2.80)	<0.001
Wealth ridit × age group	50–59: wealth-gradient slope	6.42 (5.61; 7.22)	<0.001
Wealth ridit × age group	60–74: wealth-gradient slope	9.71 (9.02; 10.40)	<0.001

Panel B. Stratum-specific SII using wealth ridits recalculated within each stratum.

Stratifier	Stratum	SII (95% CI)	p value
Sex	Women	3.87 (3.33; 4.41)	<0.001
Sex	Men	6.17 (5.83; 6.51)	<0.001
Residence	Urban	3.13 (2.74; 3.53)	<0.001
Residence	Rural	2.64 (2.05; 3.23)	<0.001
Age group	30–39	1.18 (0.97; 1.40)	<0.001
Age group	40–49	2.33 (1.85; 2.80)	<0.001
Age group	50–59	6.40 (5.59; 7.21)	<0.001
Age group	60–74	10.11 (9.38; 10.85)	<0.001

Panel C. Components of the Framingham-based metric by wealth quintile.

Variable	Q1	Q2	Q3	Q4	Q5	Q5–Q1	p value
Predicted excess cardiovascular age, years	7.14 (6.99; 7.28)	8.70 (8.53; 8.87)	9.89 (9.69; 10.09)	10.74 (10.52; 10.96)	11.25 (11.00; 11.50)	4.11 (3.82; 4.40)	<0.001
BMI, kg/m²	25.77 (25.70; 25.83)	27.85 (27.78; 27.93)	28.60 (28.51; 28.69)	28.87 (28.77; 28.96)	28.56 (28.46; 28.66)	2.79 (2.68; 2.91)	<0.001

Variable	Q1	Q2	Q3	Q4	Q5	Q5–Q1	p value
Systolic blood pressure, mmHg	121.35 (121.09; 121.62)	122.32 (122.04; 122.61)	123.39 (123.06; 123.71)	124.32 (123.95; 124.68)	124.60 (124.21; 124.99)	3.24 (2.77; 3.72)	<0.001
Current smoking, %	1.14 (1.00; 1.28)	1.62 (1.42; 1.82)	1.97 (1.73; 2.21)	2.06 (1.80; 2.32)	2.66 (2.32; 2.99)	1.51 (1.15; 1.88)	<0.001
Self-reported diabetes, %	1.98 (1.80; 2.16)	4.35 (4.02; 4.68)	6.10 (5.65; 6.54)	6.94 (6.44; 7.43)	7.57 (7.01; 8.13)	5.59 (5.00; 6.17)	<0.001
Antihypertensive treatment, %	4.24 (3.97; 4.51)	5.33 (4.97; 5.69)	6.60 (6.15; 7.05)	7.63 (7.14; 8.11)	10.02 (9.40; 10.64)	5.78 (5.10; 6.45)	<0.001

Notes: Estimates account for the ENDES complex survey design. SII, Slope Index of Inequality; BMI, body mass index; CI, confidence interval. These exploratory analyses are descriptive and should not be interpreted as causal mediation.
