# Supplementary figures and images for: Predicted Excess Cardiovascular Age and a Reverse Socioeconomic Gradient in a Middle-Income Latin American Country: A Population-Based Analysis of 163,889 Peruvians
Source: J Cardiovasc Dev Dis. 2026 Jul 9;13(7):318. doi: 10.3390/jcdd13070318 (PMC13411265; doi:10.3390/jcdd13070318)

Figure S1. Flow diagram of participant selection.

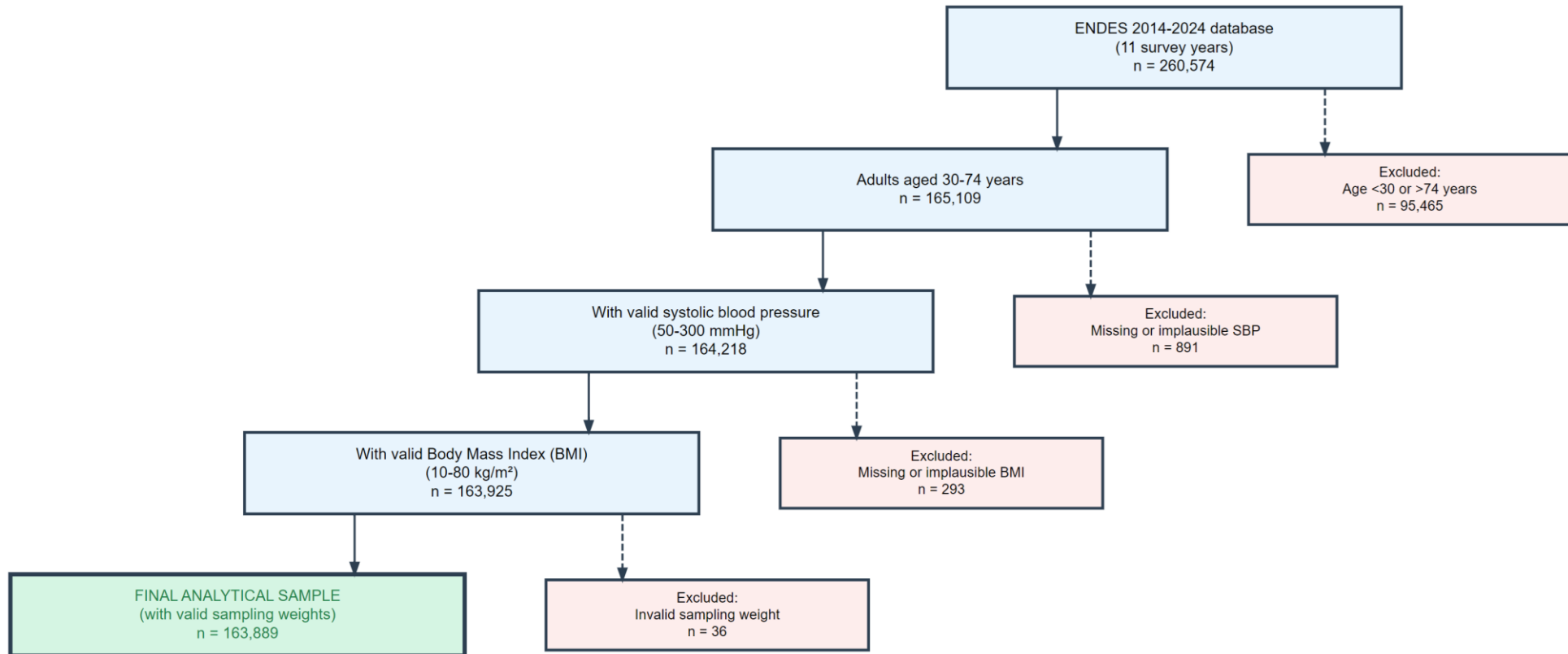

Supplement: Supplementary file 1 [file jcdd-13-00318-s001.zip › Figure S1_Flow_Diagram.pdf]

Figure S2. Socioeconomic gradient of excess cardiovascular age by sex.

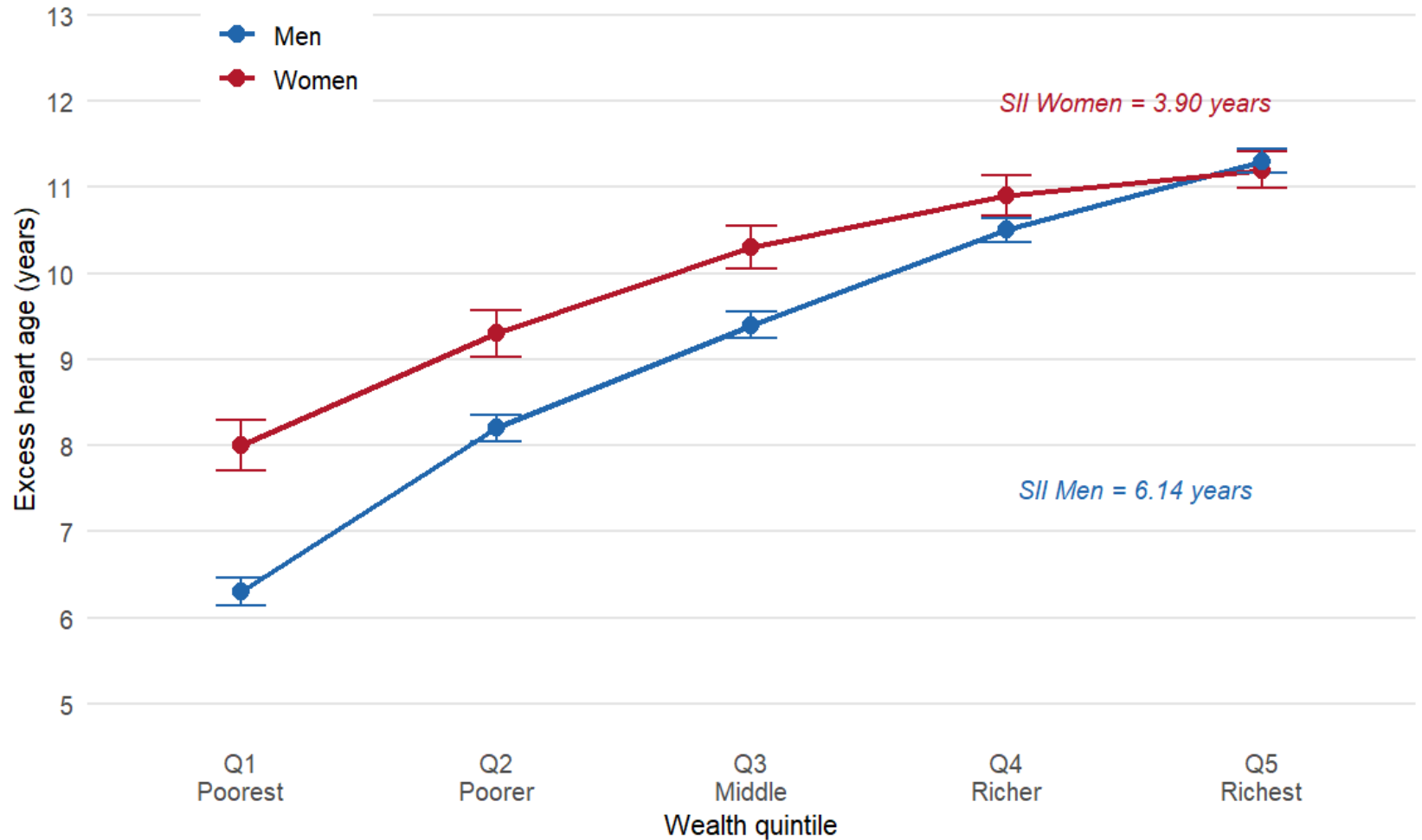

Supplement: Supplementary file 1 [file jcdd-13-00318-s001.zip › Figure S2_Socioeconomic_Gradient.pdf]

Figure S3. Proportion of adults with excess cardiovascular age  $\geq 10$  years by department.

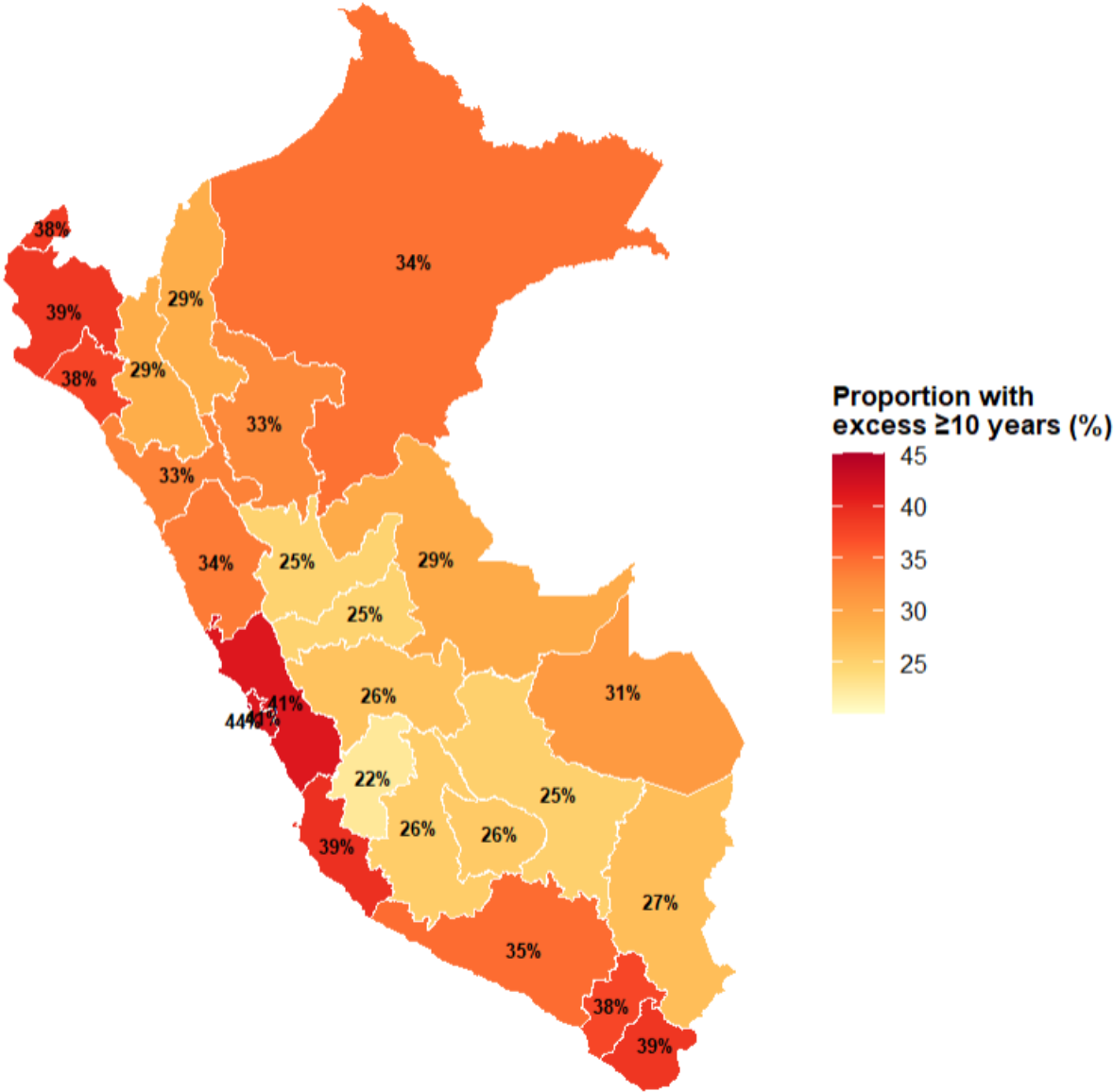

Supplement: Supplementary file 1 [file jcdd-13-00318-s001.zip › Figure S3_Proportion_10years.pdf]

Figure S5. Caterpillar plot of excess cardiovascular age by department.

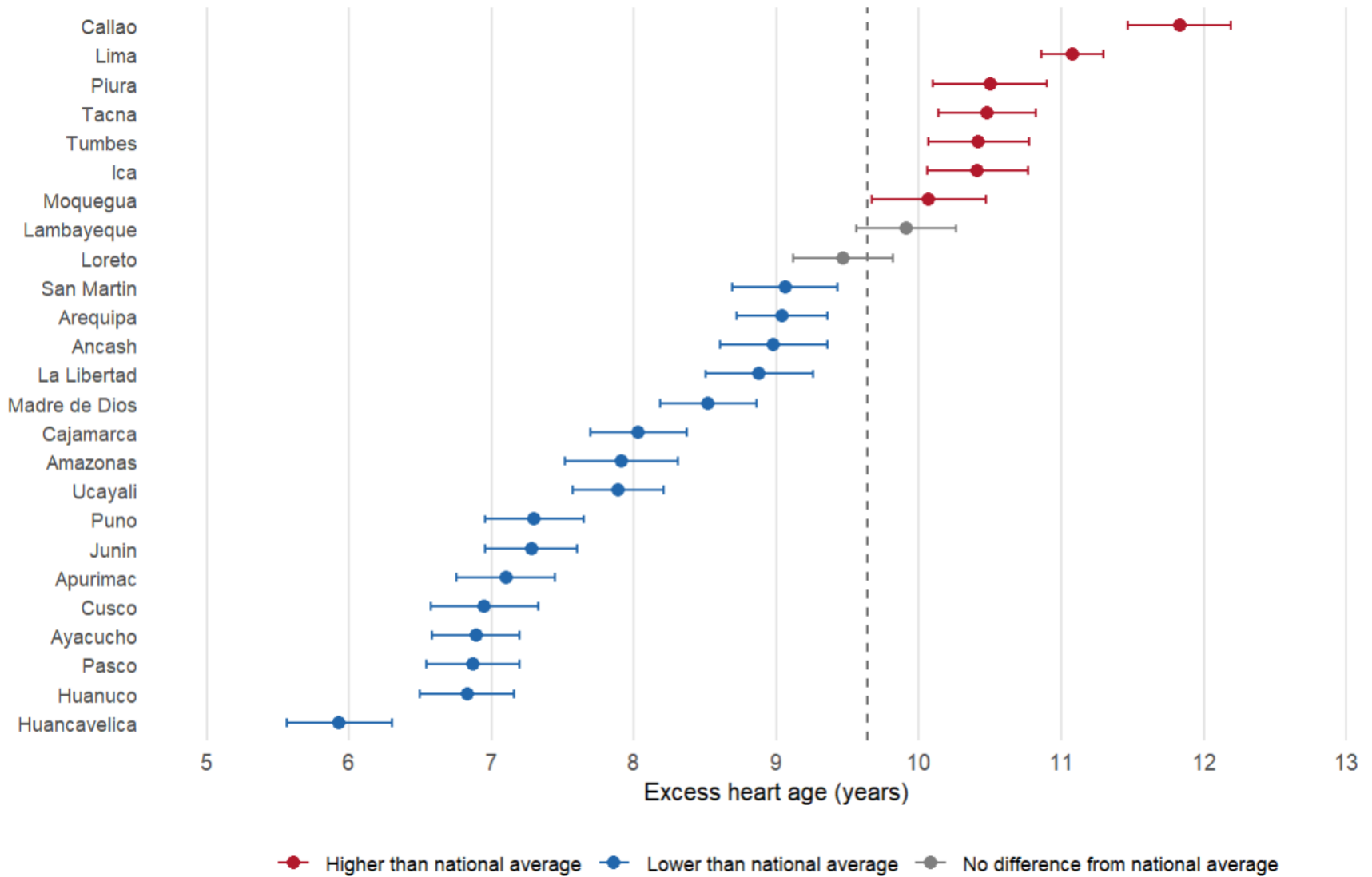

Supplement: Supplementary file 1 [file jcdd-13-00318-s001.zip › Figure S5_Caterpillar_Plot.pdf]
